# Complete Chloroplast Genome Sequence of the Long Blooming Cultivar *Camellia* ‘Xiari Qixin’: Genome Features, Comparative and Phylogenetic Analysis

**DOI:** 10.3390/genes14020460

**Published:** 2023-02-10

**Authors:** Yufen Xu, Yanju Liu, Zhaoyan Yu, Xiaocheng Jia

**Affiliations:** Hainan Key Laboratory of Tropical Oil Crops Biology, Coconut Research Institute, Chinese Academy of Tropical Agricultural Sciences, Wenchang 571339, China

**Keywords:** *Camellia* ‘Xiari Qixin’, Theaceae, chloroplast genome, phylogeny

## Abstract

The camellia flower is a famous woody plant with a long-cultivated history and high ornamental value. It is extensively planted and utilized around the world and owns a massive germplasm resource. *Camellia* ‘Xiari Qixin’ belongs to one of the typical cultivars in the four seasons camellia hybrids series. Due to its long flowering period, this kind of cultivar is identified as a precious resource of camellia flowers. In this study, the complete chloroplast genome sequence of *C.* ‘Xiari Qixin’ was first reported. Its whole chloroplast genome is 157,039 bp in length with an overall GC content of 37.30%, composed of a large single copy region (LSC, 86,674 bp), a small single copy region (SSC, 18,281 bp), and a pair of inverted repeat regions (IRs, 26,042 bp each). A total of 134 genes were predicted in this genome, including 8 ribosomal RNA genes, 37 transfer RNA genes, and 89 protein-coding genes. In addition, 50 simple sequence repeats (SSRs) and 36 long repeat sequences were detected. By comparing *C*. ‘Xiari Qixin’ and seven *Camellia* species on the chloroplast genome, seven mutation hotspot regions were identified, including *psbK*, *trnS* (GCU)-*trnG*(GCC), *trnG*(GCC), *petN*-*psbM*, *trnF*(GAA)-*ndhJ*, *trnP*(UGG)-*psaJ*, and *ycf1*. Phylogenetic analysis of 30 chloroplast genomes showed that the genetic relationship between *C.* ‘Xiari Qixin’ and *Camellia azalea* is quite close in evolution. These results could not only provide a valuable database for determining the maternal origin of *Camellia* cultivars, but also contribute to the exploration of the phylogenetic relationship and utilization of germplasm resources for *Camellia*.

## 1. Introduction

Camellia flowers, also known as tea flowers, generally refers to the flower plants of the genus *Camellia* in the family Theaceae. As one of the ten most famous flowers in China, it is also a treasure around the world, which is planted in more than 40 countries with more than 15,000 cultivars [1,2]. Horticulturalists and botanists never heisted to show their attention in every possible way. The four seasons camellia hybrids is a new generation of camellia cultivars, which is a crossing of *C*. *azalea* and traditional camellia flower cultivars. This series of cultivars is a precious camellia resource with high ornamental value. Because of their typical characteristic of flowering in all four seasons, especially in winter and spring, they are too different from traditional cultivars [3]. Simultaneously, these cultivars possess excellent cold and heat tolerance, which enables them to be cultivated in many areas. However, very few studies have focused on the molecular genetics of these cultivars. *Camellia* ‘Xiari Qixin’ is a typical representative of this cultivar group. Chinese breeders developed this cultivar by crossing *C*. *azalea* as the female parent and *C*. ‘Dr. Clifford Parks’ as the male parent. It can be treated as a very valuable landscape material for decorating gardens owing to its specific features of upright shape, dense flowers, and easy growth. Besides, this cultivar is a treasurable *Camellia* resource with strong heat tolerance, which is suitable for growth and cultivation in tropical areas. It also can be applied to improving the heat tolerance of economic plants in *Camellia*. Nevertheless, the missing molecular genetic information on *C*. ‘Xiari Qixin’ not only restricts its research on the classification and genetic diversity of the *Camellia* genus, but also impedes the application of this excellent cultivar in *Camellia* breeding.

*Camellia* spp. can be used as not only ornamental plants, but also beverage and oil plants. Their great combined economic value has long been of interest to researchers [4]. With the universal interspecific hybridization and polyploidy, *Camellia* has a high genetic heterozygosity and phenotypic polymorphism, which has made its intra-genus classification controversial for a long time and leads it to be an extremely challenging genus in plant phylogenetic investigations [5,6]. The genus *Camellia* often suffers from difficult identification, unclear parentage, and confused genetic relationship, which hinders germplasm innovation in *Camellia*. Moreover, the effective gene regions used in the phylogenetic research of *Camellia* are currently limited. Among them, the common chloroplast regions include *rpl16*, *psbA*-*trnH*, *trnL*-*F*, *matK*, etc. When these regions are applicative in research, their phylogenetic trees often have a large number of parallel branches with low bootstrap values. Due to the limited phylogenetic information, it is difficult to reflect the phylogenetic relationship among *Camellia* clearly [7,8,9,10]. However, the popular nuclear ITS region frequently utilized for phylogenetic estimations contains extensive polymorphism in this genus, precluding reliable phylogenetic assessment [11,12]. Therefore, it is necessary to develop high-resolution genetic markers to promote the species identification, germplasm screening, and phylogeny of *Camellia* to further facilitate the utilization and popularization of *Camellia* germplasm resources. The plant chloroplast genome has the characteristics of low molecular weight and simple structure. Most of them are maternal inheritance with a stable genetic ability of offspring, and there is high homology among different taxa. Meanwhile, it owns a high replicate capability, moderate nucleotide substitution rate, and difference in molecular evolution rates in various regions [13,14,15]. In recent years, chloroplast genomes have been widely investigated in plant research at different taxonomic levels, such as molecular identification [16], phylogenetics [17,18,19], phylogeography [20,21,22], evolutionary dynamics [23], and population genetics [24]. For example, Yu et al. [25] reassessed the phylogenetic relationship and temporal history of Theaceae using the chloroplast genomes of 43 species of Theaceae and discussed the development history of East Asian subtropical evergreen broadleaved forests.

In this study, based on the Illumina Hiseq2500 platform, we obtained the complete chloroplast genome of *C*. ‘Xiari Qixin’ and compared it with seven *Camellia* species. The objectives of this study were to: (1) characterize the chloroplast genome of *C*. ‘Xiari Qixin’; (2) identify the different hotspots in the eight chloroplast genome sequences; and (3) explore the phylogenetic relationship of *C*. ‘Xiari Qixin’. This study provides genetic resources for *Camellia* research on further DNA barcoding, phylogeny, and evolution.

## 2. Materials and Methods

### 2.1. Sampling

The samples of *C.* ‘Xiari Qixin’ were collected from the Germplasm Repository of Oil Camellia in Coconut Research Institute of Chinese Academy of Tropical Agricultural Sciences (CATAS) (Hainan, China; Coordinates: 19°32′4.64″ N, 110°45′47.42″ E). The total genomic DNA was extracted from leaf material with a modified CTAB method [26].

### 2.2. Sequencing

With the insert length of 350 bp, the library was constructed by the NEBNext^®^Ultra™ II DNA Library Prep Kit. Paired-end sequencing with the reads of 150 bp was conducted on the second-generation sequencing platform of Illumina Hiseq2500, and approximately 8.53 GB raw reads was totally generated. The raw data was uploaded to NCBI (available online: https://www.ncbi.nlm.nih.gov/ (accessed on 26 April 2021)), BioProject, and BioSample, and SRA numbers are PRJNA725044, SAMN18868764 and SRR14352139, respectively.

### 2.3. Chloroplast Genome Assembly and Annotations

Setting *C. pubicosta* (NC_024662.1) as the reference, the toolkit GetOrganelle [27] applied to de novo assemble the chloroplast genome. As with gene annotation, the starting position of the chloroplast genome and the IR region were determined by using online annotation software such as Geseq [28] and CpGAVAS [29]. Finally, after manually checking the annotations, the complete chloroplast genome of *C.* ‘Xiari Qixin’ was submitted to GenBank (MZ004951). The chloroplast genome was online mapped by OGDRAW v1.3.1 [30].

### 2.4. Repeat Sequences Detection

The online software MISA [31] recognized single sequence repeats (SSRs) in the chloroplast genome, and SSRs were known as microsatellites. The minimum repeat thresholds of 10, 6, 5, 5, 5, and 5 were set for mono-, di-, tri-, tetra-, penta-, and hexanucleotide repeating units, respectively. In addition, the long repeats in the chloroplast genome were detected by the online program REPuter [32], including forward, palindromic, reverse, and complement repeats. The minimum repeat size was set as 30 bp and the Hamming distance was three.

### 2.5. Codon Usage

The coding sequence (CDS) of the chloroplast genome protein-coding genes (PCGs) was extracted using PhyloSuite v1.2.2 [33]. Moreover, the amino acid composition and relative synonymous codon usage (RSCU) were predicted by CodonW v1.4.2.

### 2.6. Chloroplast Genome Comparison

*C.* ‘Xiari Qixin’ and seven common ornamental *Camellia* species, including *C*. *azalea* (KY856741), *C*. *japonica* (KU951523), *C*. *chekiangoleosa* (MG431968), *C*. *pitardii* (KF156837), *C*. *reticulata* (KJ806278), *C*. *crapnelliana* (KF753632), and *C*. *sasanqua* (MH782189), were applied for chloroplast genome comparison. The online software mVISTA [34] analyzed the difference in the eight chloroplast genome sequences with *C*. *pubicosta* (NC_024662.1) as a reference and the Shuffle-LAGAN alignment mode. Eight *Camellia* chloroplast genome sequences were aligned by MAFFT v7.475 [35]. Nucleotide diversity (π) was calculated in DnaSP v6.12.03 [36] with a window length of 600 bp and a step size of 200 bp (step size). The online software IRscope (available online: https://irscope.shinyapps.io/irapp/ (accessed on 7 March 2022)) [37] determined the structure partition boundaries of the chloroplast genome.

### 2.7. Phylogenetic Analysis

Phylogenetic analysis was carried out on *C.* ‘Xiari Qixin’, 33 *Camellia* species, and two outgroups (*Pyrenaria pingpienensis* and *Tutcheria championii*), and their details were presented in Table S1. All 36 chloroplast genomes of these taxa contained both IR regions and were aligned by MAFFT v7.475. To avoid overestimating IR regions, the IRA region was removed from the alignment. The data matrix consisting of the LSS, SSC, and IRB regions was applied for maximum likelihood (ML) phylogenetic reconstruction. The nucleotide substitution model was detected by ModelFinder [38]. The ML tree was constructed through IQ-TREE v2.1.3 [39] with six Pentaphylacaceae species as outgroups. GTR+F+R5 was set as the model, and 1000 was set for bootstrap replication.

## 3. Results

### 3.1. Chloroplast Genome Features of C. ‘Xiari Qixin’

**Figure 1:**
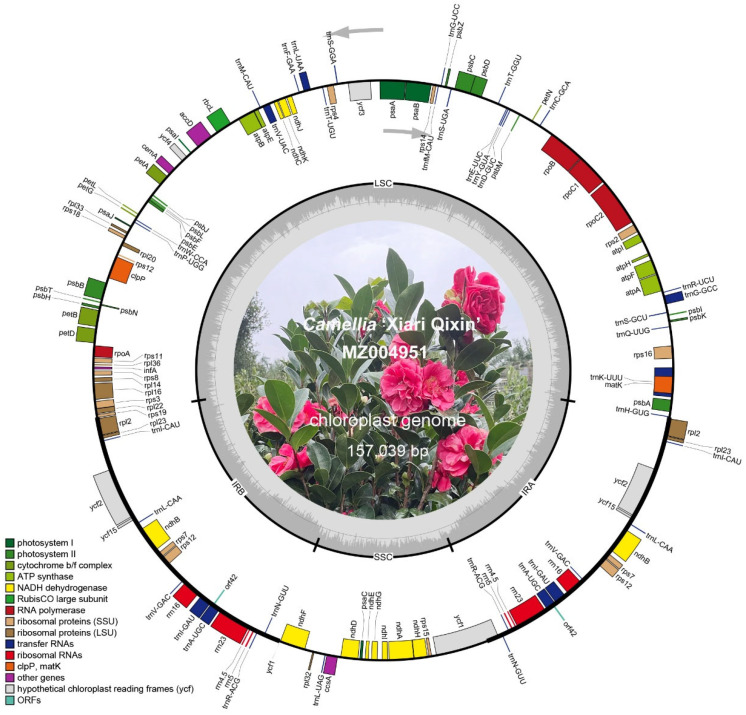
Gene map of chloroplast genome for *Camellia* ‘Xiari Qixin’. The innermost track of dark gray represents GC contents; genes shown on the outside of the map are transcribed in a clockwise direction, while genes on the inside of the map are transcribed in a counterclockwise direction; LSC, large single copy region; SSC, small single copy region; IRA/B, inverted repeat A/B region.

The chloroplast genome configurations of *C.* ‘Xiari Qixin’ and seven *Camellia* species were typically circular quadripartite (Figure 1). The eight chloroplast genomes range in size from 156,544 bp (*C*. *sasanqua*) to 157,039 bp (*C*. ‘Xiari Qixin’ and *C*. *azalea*). These genomes consisted of one large single copy region (LSC; 86,212–86,674 bp), one small single copy region (SSC; 18,234–18,406 bp), and two inverted repeat regions (IRs; 25,943–26,066 bp, each). Their total number of genes ranged from 132 (*C*. *azalea*, *C*. *japonica*, and *C*. *reticulata*) to 137 (*C*. *pitardii*), including 87–89 protein-coding genes (PCGs), 36–40 tRNAs (transfer RNA), and 8 rRNAs (ribosomal RNA). The total GC contents of these genomes were similar to each other, and its average content was calculated to be 37.30% with a bias of less than 0.05%. The average GC contents of the LSC, SSC, and IR were 35.31%, 30.57%, and 43.00%, respectively, while the GC contents of IR gave the highest result (Table 1).

According to the chloroplast genome annotation, the chloroplast genes of *C.* ‘Xiari Qixin’ could be divided into four function categories (Table S2), which are photosynthesis genes (44), self-replication genes (59), other genes (6), and unknown function genes (6). Simultaneously, there were 18 intronic genes in the chloroplast genome of *C.* ‘Xiari Qixin’, and they were distributed in three different regions, LSC (13), IR (3), and SSC (1, *ndhA*), respectively (Table S3). They contained 6 tRNAs and 12 PCGs; among them, the *trnK*-UUU intron was the longest (2496 bp), and the *trnL*-UAA intron was the shortest (523 bp). Meanwhile, there were 16 genes with only one intron, except *ycf3* and *clpP*.

### 3.2. Repeat Sequences Analysis

**Figure 2:**
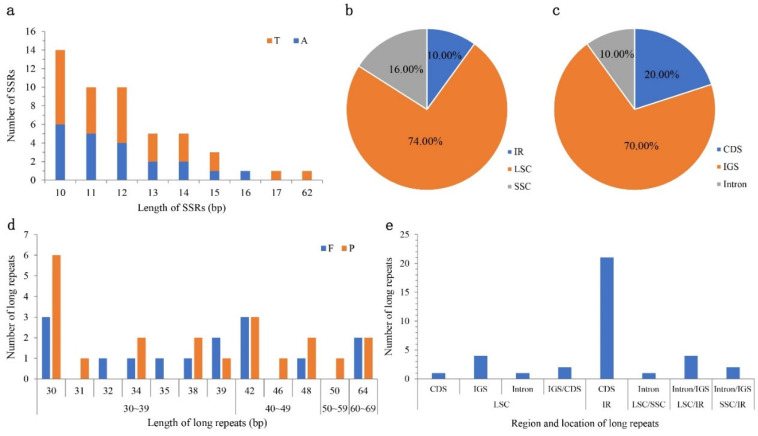
The type, frequency, and distribution of simple sequence repeats (SSRs) and long repeated sequences in the chloroplast genome of *C*. ‘Xiari Qixin’: (**a**) Frequency of different SSR repeat motifs by length. A, A base. T, T base; (**b**) Proportion of SSRs in different regions; (**c**) Proportion of SSRs in different locations. IGS: intergenic space; CDS: protein-coding regions; (**d**) Frequency of different lengths in long repeats. F: Forward repeat, P: Palindrome repeat; (**e**) Frequency of long repeats by distribution.

A total of 50 SSRs have a length of 10 to 62 bp, which could be detected and found in the chloroplast genome of *C.* ‘Xiari Qixin’ (Figure 2). They all belong to mononucleotide types with A/T base as the repeated unit. Among them, 37 SSRs were distributed in several regions involving the LSC region (37, 74.00%), the SSC region (8, 16.00%), and the IR region (5, 10.00%), in addition to 35 SSRs located in the intergenic region (70.00%), 10 SSRs in the protein-coding region (20.00%; *atpB*, *ccsA*, *rpoA*, *rpoB*, *rpoC2*, and *ycf1*), and five SSRs in introns (10.00%; *trnI*-GAU, *ycf3*, *clpP*, and *rpl6*).

A total of 36 long repeated sequences were identified to exist in the chloroplast genome of *C.* ‘Xiari Qixin’, consisting of 15 forward repeats (41.67%) and 21 palindrome repeats (58.33%) (Figure 2). The main length of repeats was 30–40 bp (58.33%). Furthermore, 21 repeats were distributed in IR (58.33%), eight repeats in LSC (22.22%), and the remaining seven repeats suspended across two structural regions (19.44%), named LSC/IR (4), LSC/SSC (1), and IR/SSC (2). Moreover, these long repeat sequences were mainly distributed in the protein-coding region (61.11%), and those were *psaB*/*psaA* (1) and *ycf2* (21), while fewer repeats were completely located in intron (5.56%).

### 3.3. Codon Usage

89 CDS were extracted from the chloroplast genome of *C*. ‘Xiari Qixin’. The total length of all detected coding regions was 74,787 bp and was encoded by 24,929 codons (Table S4). The common encoded amino acid types were determined as asparagine (Asn) and isoleucine (Ile) with 2192 (8.79%) and 2155 (8.64%) codons, respectively. The uncommon encoded amino acids were cysteine (Cys) and tryptophan (Trp) with the value of 273 (1.10%) and 437 (1.75%) codons, respectively. The RSCU values of all codons ranged from 0.32 (CGC) to 1.95 (UUA). Except for the codon UUG (Leu), the remaining 29 preferred synonymous codons (RSCU > 1) all ended with A/T (U) base. The preferred terminator used was UAA. The codon AUG (methionine, Met) and UGG (Trp) had no bias (RSCU = 1).

### 3.4. Comparative Analysis of the Eight Chloroplast Genomes of Camellia

**Figure 3:**
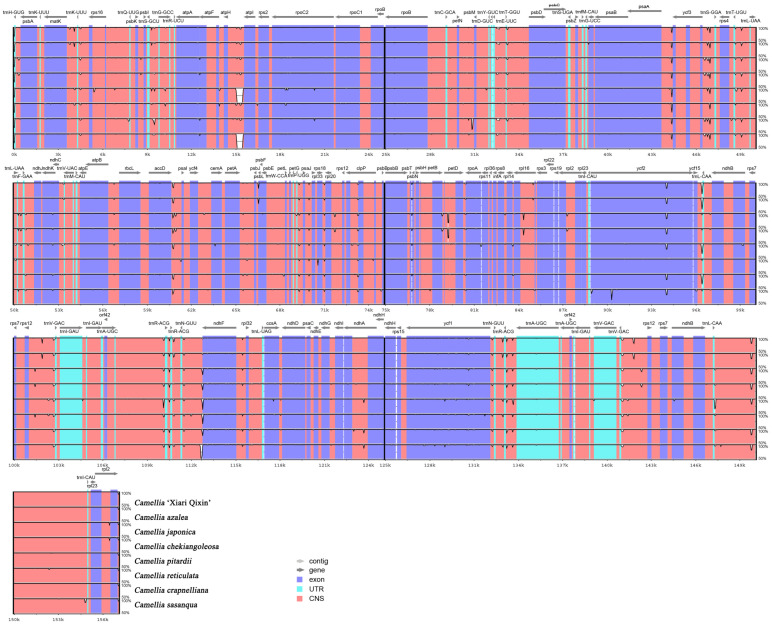
Global alignment of chloroplast genomes for *C*. ‘Xiari Qixin’ and seven *Camellia* species. The vertical scale represents the degree of identity, ranging from 50% to 100%. Coding and non-coding regions are colored in blue and red, respectively. Gray arrows indicate the position and direction of each gene.

Sequence identity analysis of mVISTA revealed that the overall chloroplast genomes of *C*. ‘Xiari Qixin’ and seven *Camellia* species were highly similar to each other, and only a few regions had a sequence identity lower than 90% (Figure 3). It indicated that these *Camellia* chloroplast genomes were quite conserved. As shown in Figure 3, the IR region was more conservative than the LSC and SSC regions. The divergence degree of the coding region was lower than the non-coding region. Furthermore, in the coding region, the *ycf1* and the *ycf2* genes were significantly different, while the other genes were relatively alike. In the non-coding region, there were great differences among intergenic regions, such as *atpH*-*atpI*, *ycf3*-*trnS* (GGA), *accD*-*psaI*, and *ycf15*-*trnL* (CAA).

**Figure 4:**
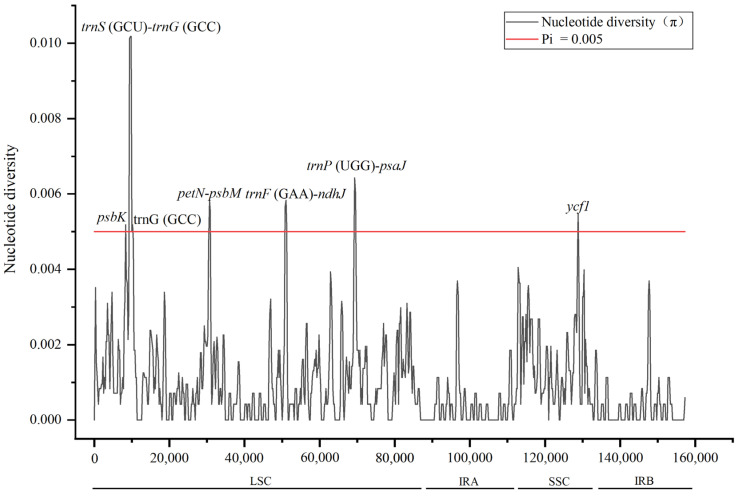
Nucleotide diversity of chloroplast genomes for *C*. ‘Xiari Qixin’ and seven *Camellia* species. The *x*-axis represents the base sequence of the alignment, and the *y*-axis represents the nucleotide diversity (π) value.

The DnaSP sliding window was applicated for detecting highly variable regions among eight *Camellia* chloroplast genomes. As shown in Figure 4, the range of nucleotide diversity (π) was found from 0.00000 to 0.01018, with the average value of 0.00097. The IR region was less variable than the LSC and SSC regions. Seven sequence mutation hotspots (π > 0.005) were identified, of which six were located in the LSC, including *psbK*, *trnS* (GCU)-*trnG* (GCC), *trnG* (GCC), *petN*-*psbM*, *trnF* (GAA)-*ndhJ*, *trnP* (UGG)-*psaJ*, and one placed in the SSC (*ycf1*). At the same time, except *psbK*, *trnG* (GCC), and *ycf1* located in the coding region, the other four fragments were distributed in intergenic regions of the non-coding region. The highest degree of nucleotide diversity in the coding region and non-coding region was *ycf1* and *trnS* (GCU)-*trnG* (GCC), respectively.

### 3.5. IR Expansion and Contraction

**Figure 5:**
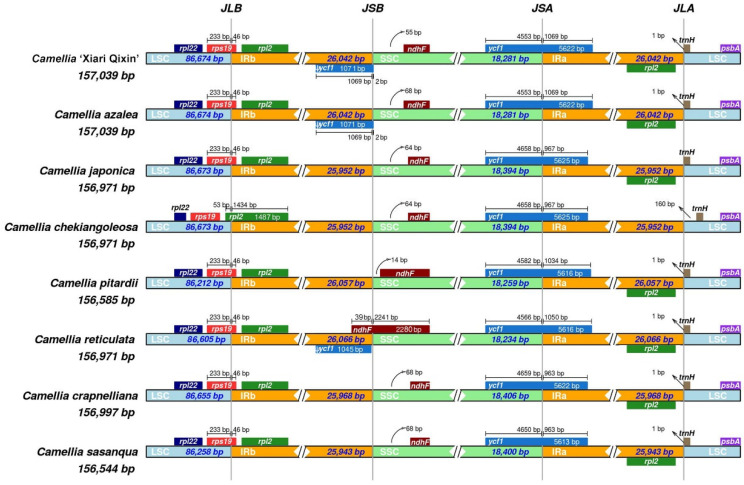
Comparison of structure borders among eight chloroplast genomes from *C*. ‘Xiari Qixin’ and seven *Camellia* species. JLB (IRB/LSC), JSB (IRB/SSC), JSA (SSC/IRA), and JLA (IRA/LSC) represent the junction between each corresponding two regions on the genome; genes transcribed by the positive strand are shown on top of line whereas genes that are transcribed by the negative strand are shown on below line.

The boundary comparison of IR and LSC/SSC among the eight chloroplast genomes was shown in Figure 5. At the LSC/IRB boundaries, except for *C*. *chekiangoleosa,* located in *rpl2*, the other seven chloroplast genomes were distributed within *rps19*, with 233 bp of these *rps19* genes located at the LSC and 46 bp extended to the IRB. The SSC/IRB boundaries of the eight chloroplast genomes had a great variation. The *ndhF* of *C*. *reticulata* crossed the boundary and 39 bp entered the IRB region. However, the SSC/IRB boundaries of the other seven chloroplast genomes were 14 (*C*. *pitardii*)–68 bp (*C*. *azalea*, *C*. *crapnelliana*, and *C*. *sasanqua*) away from *ndhF,* located in the SSC. In addition, those boundaries of *C*. ‘Xiari Qixin’ and *C*. *azalea* were located within the *ycf1* pseudogene. The *ycf1* gene crossed SSC/IRA boundary. These *ycf1* genes distributed 4553 (*C*. ‘Xiari Qixin’ and *C*. *azalea*) to 4659 bp (*C*. *crapnelliana*) into the SSC and 963 (*C*. *crapnelliana* and *C*. *sasanqua*) to 1069 bp (*C*. ‘Xiari Qixin’ and *C*. *azalea*) into the IRA. At the IRA/LSC junction, the *trnH* was located entirely in the LSC, away from zero (*C*. *japonica*) to 160 bp (*C*. *chekiangoleosa*). However, the copy gene of *rpl2* was completely preserved in the IRA except for *C*. *chekiangoleosa*.

### 3.6. Phylogenetic Analysis

**Figure 6:**
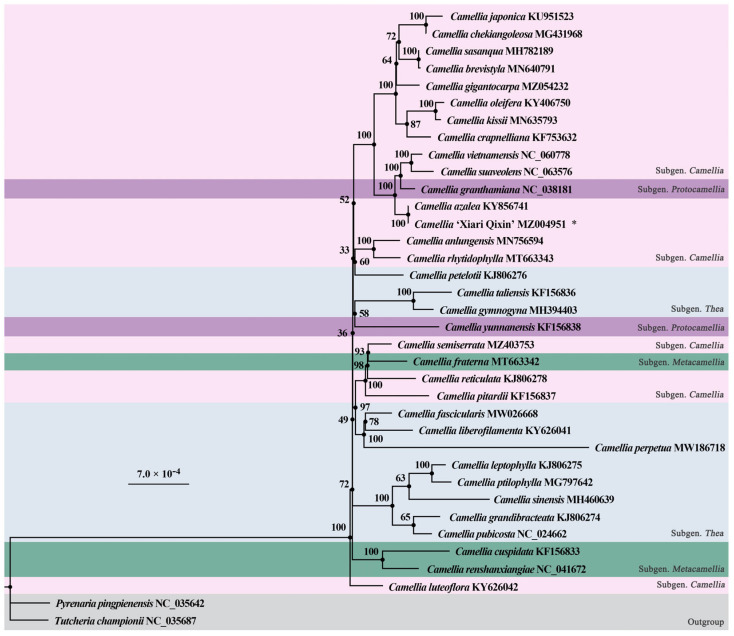
Phylogenetic tree of 36 chloroplast genomes inferred from maximum likelihood (ML). The bootstrap values are marked respectively at each branch node. *, the sample was sequenced and reported in this research.

In order to understand the phylogenetic position of *C*. ‘Xiari Qixin’ in the *Camellia*, *C*. ‘Xiari Qixin’ and 33 *Camellia* species were applied for phylogenetic analysis with two Theaceae species as outgroups (Figure 6). All 34 *Camellia* plants were gathered in a monophyletic branch with BS = 100. The topological configuration of the phylogenetic tree was relatively stable, and the parallel structure was only observed in *C*. ‘Xiari Qixin’ and *C*. *azalea*, while the remaining 32 species of the *Camellia* genus were single branches to each other. The four subgenera of *Camellia* were all polyphyletic groups; among them, the plants in Subgen. *Camellia* were clustered into three different branches, which were located in the base, middle, and upper parts of the tree. *C*. ‘Xiari Qixin’ was distributed in the Subgen. *Camellia* of the top branch. Moreover, it was closely related to four species, including three in Subgen. *Camellia* (C. *azalea*, *C*. *suaveolens*, and *C*. *vietnamensis*) and one in Subgen. *Protocamellia* (*C*. *granthamiana*). In addition to that, this cultivar had the nearest evolutional relationship with *C*. *azalea*.

## 4. Discussion

The *C*. ‘Xiari Qixin’ chloroplast genome structure is a typical circular quadripartite, which is alike to most angiosperms. The previous research mentioned that the size of chloroplast genomes in angiosperms is determined to be from 120 to 160 kb [40]. In this study, the lengths of chloroplast genomes in *C*. ‘Xiari Qixin’, seven *Camellia* species, and previous *Camellia* plants [6,41,42,43] are similar to each other, within 1 kb deviation, ranging from 156,544 to 157,353 bp. It is generally believed that the expansion/contraction of the IR boundary of the chloroplast genome is one of the main reasons causing the difference in the size of chloroplast genomes in angiosperms [44]. In the comparative analysis of structure boundaries of the chloroplast genomes, slight variations were detected in the IR/SC boundary positions among *C*. ‘Xiari Qixin’ and seven *Camellia* species, which is consistent with the former observation in the *Camellia* genus [42]. Expansion of the IR boundary was only observed in *rpl2* of *C*. *chekiangoleosa* and *ndhF* of *C*. *reticulate*. In addition, the extension of the *rpl2* gene was also found in *Ulmus* [45], *Manglietia* [46], and *Physalis* [47]. To further verify variation of the chloroplast genome, synteny analysis was carried out on *C*. ‘Xiari Qixin’ and seven *Camellia* species by MAUVE [48] (Figure S1). However, the eight chloroplast genomes had high collinearity, but no gene rearrangement and inversion occurred. Of course, the expansion/contraction of the IR region detected initially in this research, as well as other yet unobserved structural variations in the chloroplast genome, such as gene replication, gene inversion, and so on, need to be further checked and verified using biological replicates of multiple individuals.

Repetitive sequences and SSRs are widely distributed in the chloroplast genome, and both are closely related to genome rearrangement and recombination. They are important molecular markers and are extensively used in plant population genetics and phylogeny [49,50]. This study identified 50 SSRs in the chloroplast genome of *C*. ‘Xiari Qixin’, and all of these belong to the mononucleotide type. They were composed of bases A/T, and most of them were distributed in the LSC. These results are similar to the SSR analysis of other *Camellia* chloroplast genomes [51,52]. Furthermore, the detected long repeats and SSRs can provide molecular marker information to reveal the population-level polymorphism and phylogeny of *Camellia* in the future research.

Mutation, genetic drift, and natural selection are the influencing factors of codon preference. Moreover, the codon usage analysis can help scholars understand the evolution process [53]. The result of codon analysis for *C*. ‘Xiari Qixin’ showed that 96.67% of the 30 codons (RSCU > 1) ended with A/T (U) base, which indicated that the chloroplast genes of *C*. ‘Xiari Qixin’ prefer to end with A/T base. This is consistent with previous observations in the chloroplast genomes of other angiosperms [54,55]. Knight et al. [56] developed a model of the nucleotide-action process to explain codon usage, and their model indicated that the GC base composition of the genome drives codon usage. Nevertheless, some scholars considered that the genome-wide codon bias is determined by the non-random mutation of the genome and the selective force of protein translation efficiency [57,58]. Hence, this research speculates that most codons in the chloroplast genome end in A/T base, probably caused by the base mutation preference for A/T base and the role of selection pressure. In addition, this result can help to deepen the comprehension of the genetic structure of *C*. ‘Xiari Qixin’ and provide a reference for the technical research on chloroplast genetic engineering to improve the meaningful traits of tea flowers.

Plant molecular systematics and DNA barcoding largely depend on chloroplast gene sequences [59]. The comparison of the whole chloroplast genome sequences between *C*. ‘Xiari Qixin’ and seven related species exhibited a high similarity. Their coding regions were more conservative than non-coding regions, similar to other angiosperms [60,61]. Seven mutated hotspots were identified by nucleotide diversity analysis, and some of them can also be observed in other angiosperms, such as *trnS*(GCU)-*trnG*(GCC) [62], *petN*-*psbM* [63], *trnP*(UGG)-*psaJ* [64], and *ycf1* [61]. These regions are likely to undergo a faster nucleotide substitution at the species level, providing important references for the development of DNA barcodes. In addition, the chloroplast genome is hundreds of times longer than that of the common barcode sequence with abundant variation sites, which can be considered as a super barcode for species identification [65]. To sum up, the identified mutation hotspots and sequenced chloroplast genome in our study are potential molecular marker tools, which can provide a large number of informative sites for the phylogeny and molecular identification of the genus *Camellia*.

Since the chloroplast genome is haploid and maternally inherited, its offspring are genetically stable [66]. More and more scholars would like to apply it to the in-depth study of the genetic background of cultivars. Wu et al. [67] evaluated the genetic resources of *C*. *oleifera* cultivars based on the chloroplast genome. Xu et al. [68] explored the maternal origin of modern *Chrysanthemum* cultivars using the chloroplast genomes of 36 chrysanthemum cultivars and some related species. We constructed a phylogenetic tree from 30 chloroplast genomes, and found that *C*. ‘Xiari Qixin’ has the closest relationship with its female parent *C*. *azalea*. Thirty-four *Camellia* plants clustered into a monophyletic group, which is consistent with the research results of Yu et al. [25]. Currently, the taxonomic system of Zhang Hongda in *Flora of China* is widely applied for the classification of the *Camellia* genus in China, and the genus is divided into four subgenera, 20 sections, and 280 species in this system. However, our phylogenetic analysis was unable to demonstrate that these four subgenera are monophyletic groups. Consequently, it is necessary to combine traditional morphological classification, molecular markers, and more genomic data and resolve the controversy on *Camellia* taxonomy in future research. Taking advantage of the chloroplast genome, our research has proven that *C*. *azalea* is the parental source of *C*. ‘Xiari Qixin’, which is consistent with the objective situation of *C*. ‘Xiari Qixin’ cultivation. This suggests that the chloroplast genome can be developed as an effective tool for the maternal identification of hybrid species or cultivars of *Camellia*, which is conducive to a better evaluation of the genetic relationship in the *Camellia* genus. In the future, the evolution and development of cultivated *Camellia* spp. can be explored more systematically and comprehensively by utilizing the chloroplast genome and nuclear genome, combining with more species and cultivar taxa of *Camellia*.

## 5. Conclusions

In this study, the complete chloroplast genome of *C*. ‘Xiari Qixin’ was first reported, which enriched the genetic resources and laid the foundation for exploring its genetic background and resource utilization. More specifically, its structure, gene composition, GC content, and codon bias were alike to those of typical angiosperms. Similarly, the chloroplast genomes of *C*. ‘Xiari Qixin’ possessed some common characters with seven *Camellia* species such as size, structure, gene composition, and low sequence variation, which proved that the chloroplast genome of *Camellia* is relatively conservative. Additionally, seven mutated hotspots were identified by comparison, and they can be applied in the molecular identification and phylogenetic development of *Camellia*. The phylogenetic results exhibited the closest genetic relationship between the hybrid camellia cultivar *C*. ‘Xiari Qixin’ and its female parent, *C*. *azalea*. In summary, these results contribute to tracing the maternal parentage of *Camellia* cultivars and providing a better understanding of the phylogeny and genetic improvement of *Camellia* germplasm resources.

## Figures and Tables

**Table 1:** The features of chloroplast genomes for *C.* ‘Xiari Qixin’ and seven *Camellia* species.

Species		*C.* ‘Xiari Qixin’	*C. azalea*	*C. japonica*	*C. chekiangoleosa*	*C. pitardii*	*C. reticulata*	*C. crapnelliana*	*C. sasanqua*
GenBank number		MZ004951	KY856741	KU951523	MG431968	KF156837	KJ806278	KF753632	MH782189
Length (bp)	Total	157,039	157,039	156,971	156,971	156,585	156,971	156,997	156,544
	LSC	86,674	86,674	86,673	86,673	86,212	86,605	86,655	86,258
	SSC	18,281	18,281	18,394	18,394	18,259	18,234	18,406	18,400
	IR	26,042	26,042	25,952	25,952	26,057	26,066	25,968	25,943
Gene number (unique)	Total	134 (115)	132 (115)	132 (111)	135 (116)	137 (117)	132 (115)	136 (116)	135 (115)
	PCGs	89 (81)	87 (81)	88 (78)	88 (82)	89 (82)	87 (81)	89 (82)	88 (81)
	tRNA	37 (30)	37 (30)	36 (29)	39 (30)	40 (31)	37 (30)	39 (30)	39 (30)
	rRNA	8 (4)	8 (4)	8 (4)	8 (4)	8 (4)	8 (4)	8 (4)	8 (4)
GC content (%)	Total	37.30	37.30	37.29	37.29	37.34	37.31	37.30	37.32
	LSC	35.30	35.30	35.28	35.28	35.35	35.32	35.30	35.33
	SSC	30.57	30.57	30.53	30.54	30.60	30.57	30.60	30.58
	IR	42.98	42.98	43.03	43.03	42.98	42.97	43.01	43.03

Note: LSC, large single copy region; SSC, small single copy region; IR, inverted repeat region; PCGs, protein-coding genes; tRNA, transfer RNA; rRNA, ribosomal RNA.

## Data Availability

The genome sequence of *C*. ‘Xiari Qixin’ are openly available in NCBI (GenBank accession number: MZ004951). The associated BioProject, Bio-Sample, and SRA numbers are PRJNA725044, SAMN18868764, and SRR14352139 respectively.

## Supplementary Materials

The following are available online at https://www.mdpi.com/article/10.3390/genes14020460/s1, Table S1: Details of the species selected for the ML tree. Table S2: Genes identified in the chloroplast genome of *C*. ‘Xiari Qixin’. Table S3: Genes with introns in the *C*. ‘Xiari Qixin’ chloroplast genome, including the exon and intron length. Table S4: Relative synonymous codon usage of protein coding region in the *C*. ‘Xiari Qixin’ chloroplast genome. Figure S1: Collinearity analyses on chloroplast genomes of *C*. ‘Xiari Qixin’ and seven *Camellia* species. Within each of the MAUVE alignments, local collinear blocks are represented by blocks of the same color connected by lines.
