# Supplementary figures and images for: Complete Chloroplast Genome Sequence of the Long Blooming Cultivar Camellia ‘Xiari Qixin’: Genome Features, Comparative and Phylogenetic Analysis
Source: Genes (Basel). 2023 Feb 10;14(2):460. doi: 10.3390/genes14020460 (PMC9956581; doi:10.3390/genes14020460)

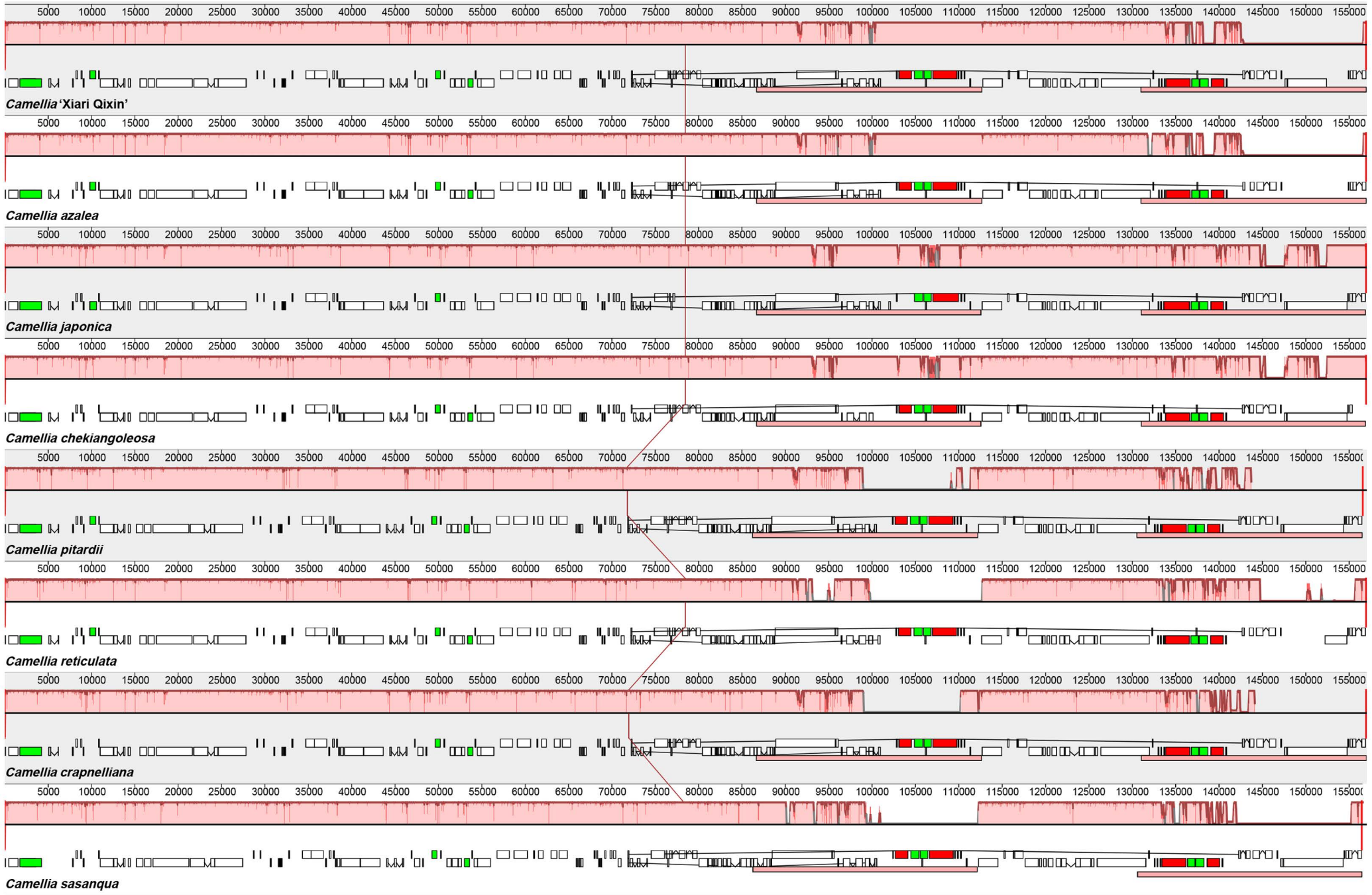

Supplement: Supplementary file 1 [file genes-14-00460-s001.zip › Figure_S1.pdf]
